# Correlation between PPAR Gene Polymorphisms and Primary Nephrotic Syndrome in Children

**DOI:** 10.1155/2013/927915

**Published:** 2013-09-11

**Authors:** Jiaping Jin, Guixia Ding, Huaying Bao, Ying Chen, Yuan Han, Fei Zhao, Songming Huang, Aihua Zhang

**Affiliations:** ^1^Department of Nephrology, Nanjing Children's Hospital, Nanjing Medical University, 72 Guangzhou Road, Nanjing, Jiangsu 210008, China; ^2^Institute of Pediatrics, Nanjing Medical University, Nanjing, Jiangsu 210029, China

## Abstract

Pediatric primary nephrotic syndrome (PNS) is a chronic disease promoted by metabolic and immune dysfunctions. Peroxisome proliferator-activated receptor (PPAR) polymorphisms have been associated with a variety of metabolic and kidney disorders. We therefore hypothesized that PPAR polymorphisms might be involved in the pathophysiology of PNS. We compared the distributions of the PPAR-*γ* Pro12Ala and Val290Met, PPAR-*γ* coactivator-*α* (PGC-1*α*) Gly482Ser, and PPAR-*α* Leu162Val single nucleotide polymorphisms (SNPs) between children with PNS and normal controls and analyzed their correlations with clinical and metabolic indicators and steroid responsiveness. There were no significant differences in distributions of any of the polymorphisms between PNS cases and controls. However, PNS patients with the PPAR-*γ* (Pro12Ala) PP genotype had significantly higher fasting serum insulin, IgA, and HOMA-IR levels and lower insulin sensitivity than did patients with PA and AA genotypes. Additionally, the PGC-1*α* (Gly482Ser) A allele was associated with lower CD8+ T-cell counts and higher triglyceride and complement C3 levels compared with the G allele. No polymorphisms were related to hormone sensitivity. These results suggest that the PPAR-*γ* (Pro12Ala) and PGC-1*α* (Gly482Ser) SNPs may influence insulin and triglyceride metabolism in children with PNS and may thus be relevant to the prognosis of this chronic condition.

## 1. Introduction

Peroxisome proliferator-activated receptors (PPARs) are a group of ligand-activated nuclear transcription factors belonging to the type II nuclear receptor superfamily. Three PPAR subtypes, PPAR-*α*, PPAR-*β*, and PPAR-*γ*, have been identified in amphibians, rodents, and humans [1]. Recent studies have shown associations between PPAR gene polymorphisms and metabolic syndrome (MS) and the development of insulin resistance (IR). Gouda et al. [2] performed a meta-analysis of 32,849 patients with type II diabetes mellitus (T2DM) and 47,456 normal controls and found that the PPAR-*γ* (Pro12Ala) gene polymorphism was associated with IR and T2DM. In addition, Sparsø et al. [3] showed that the PPAR-*α* (Leu162Val) gene polymorphism was associated with obesity, T2DM, and abnormal lipid metabolism, while Andrulionytè et al. [4] found a link between the PPAR-*γ* coactivator-*α* (PGC-1*α*) Gly482Ser gene polymorphism and conversion from impaired glucose tolerance to T2DM.

Children with primary nephrotic syndrome (PNS) suffer from metabolic abnormalities including glycolipid disorders, altered hemodynamics, and immune dysfunction, and previous studies have demonstrated that these disorders promote the progression of renal diseases and the development of end-stage renal disease (ESRD) [5]. Furthermore, the Ala allele of PPAR-*γ* was shown to be associated with a reduced glomerular filtration rate (GFR) and increased occurrences of ESRD, cardiovascular events, and mortality in patients with diabetic nephropathy [6]. We therefore hypothesized that PPAR gene polymorphisms may be associated with the occurrence, clinical manifestations, pathological type, and treatment response in patients with PNS. A better understanding of these relationships may provide a theoretical basis for further studies of the pathophysiological role of PPARs in PNS. 

 We tested this hypothesis using clinical data from children with PNS and from healthy children (normal controls, NCs). The distributions of the single nucleotide polymorphisms (SNPs) Pro12Ala and Val290Met in the PPAR-*γ* gene, Gly482Ser in the PGC-1*α* gene, and Leu162Val in the PPAR-*α* gene were determined in PNS and NC children. In addition, the associations between these polymorphisms and clinical metabolic indicators, proteinuria, renal pathology, and treatment response in patients with PNS were examined to investigate the pathophysiological role of PPARs in PNS and to determine the potential role of these polymorphisms in treatment planning and prognosis determination in children with PNS. 

## 2. Subjects and Methods

### 2.1. Subjects

Patients with a diagnosis of PNS treated at the Nanjing Children's Hospital, China, between July 2008 and November 2010 were evaluated. Genotype determinations for Pro12Ala and Val290Met of the PPAR-*γ* gene and Leu162Val of the PPAR-*α* gene were performed in 111 PNS patients (80 male, 31 female; mean age 3.33 years, range 0.67–13.08). Genotype determination for Gly482Ser of the PGC-1*α* gene was performed in 108 patients (78 male, 30 female; mean age 3.47 years, 0.67–13.08). All subjects were diagnosed with PNS according to the diagnostic criteria outlined by the clinical classification diagnosis and treatment of glomerular disease in children [7]. The presence of secondary kidney disease was excluded. None of the included patients were receiving *β*-blockers, diuretics, calcium-channel blockers, angiotensin-converting enzyme inhibitors, angiotensin II receptor blockers, glucocorticoids, or other immunosuppressants at the time of inclusion. 

 All patients were followed for between 4 months and 2.5 years, and the duration of followup reflected the response to glucocorticoids. Patients who received steroids were divided into three groups: steroid-responsive nephrotic syndrome (NS), where urine was negative for protein within 8 weeks of treatment (*n* = 83), steroid-resistant NS, where urine still contained protein at 8 weeks (*n* = 13), and steroid-dependent NS, where urinary protein reappeared after the steroid dose was reduced (*n* = 12). 

NCs were recruited from patients admitted to the hospital for elective surgery. Genotype determinations for Pro12Ala and Val290Met of the PPAR-*γ* gene and Leu162Val of the PPAR-*α* gene were performed in 111 healthy children who received a physical examination at our hospital (94 male, 17 female; mean age 3.5 years, range 0.80–10.75). Genotype determination for Gly482Ser of the PGC-1*α* gene was performed in 110 NCs (93 male, 17 female; mean age 3.54 years, range 0.80–10.75). A detailed check of medical history was performed to exclude children who had been premature and of low birth weight, had macrosomia, or whose mother had gestational diabetes mellitus or any other significant conditions. Age, sex, and body mass index (BMI) did not differ significantly between the NC and PNS children.

 The local ethics committee approved the study, and informed consent was obtained from all participating children and their parents.

### 2.2. Detection of SNP Positions and Sequencing of PCR Products

**Figure 1:**
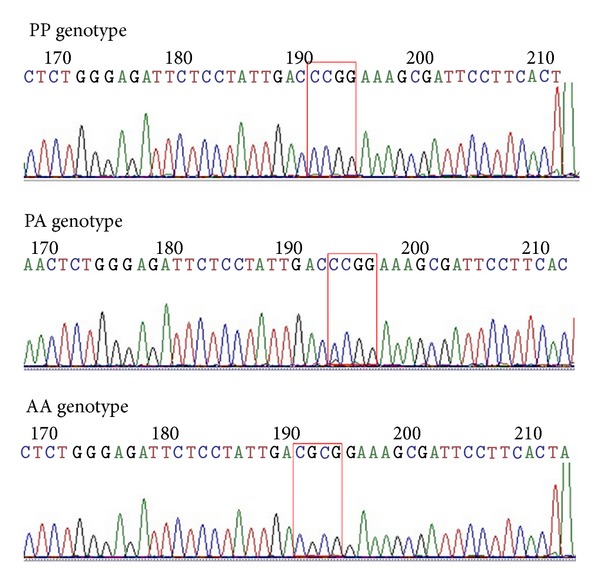
The sequencing map of the PCR amplification product with the PP genotype, the PA genotype, and the AA genotype of the PPAR-*γ* gene at the Pro12Ala position.

**Figure 2:**
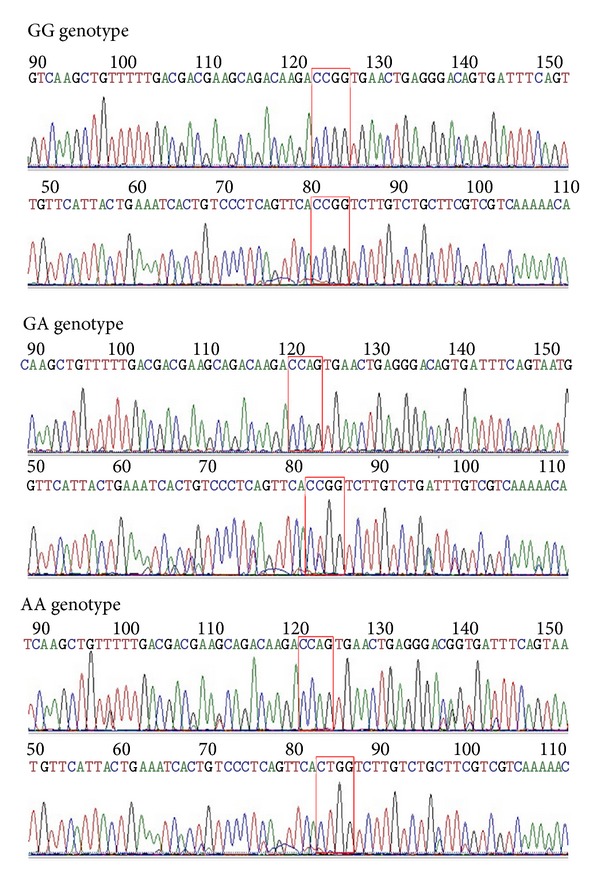
The sequencing map of the PCR amplification product for the GG genotype, the GA genotype, and the AA genotype of the PGC-1*α* gene at the Gly482Ser position.

DNA was extracted from peripheral venous blood using a genomic DNA purification kit (Qiagen Sciences, Germantown, MD, USA). The SNP genotypes were examined using polymerase chain reaction (PCR) restriction fragment length polymorphism. The primers were synthesized by Invitrogen, and the sequences were as follows: PPAR-*γ* Pro12Ala sense, 5′-CAAGCCCAGTCCTTTCTGTG-3′; antisense, 5′-GCCTTTCG CTAAGGAAGTGA-3′; PPAR-*γ* Val290Met sense, 5′-ATTCCTT AATGATGGGAGAA-3′; antisense, 5′-TCCTCTAGTGTCTCATACGGTT-3′; PPAR-*α* Leu162Val sense, 5′-GACTC AAGCTGGTGTATGACAAGT-3′; antisense, 5′-TAAGACAGCCTTACAGTGTGTTGC-3′; and PGC-1*α* Gly482Ser sense, 5′-TGCTACCTGAGAGAG ACTTTG-3′; antisense, 5′-CTTTCATCTTCGCTGTCATC-3′. The cycling conditions were 95°C for 5 min followed by 30 cycles of 94°C for 30 s, 55°C for 40 s, and 72°C for 30 s. The PCR product for the Pro12Ala position of the PPAR-*γ* gene was 237 bp in size. Following digestion with the restriction endonuclease Hpa II, the genotypes included PP (217 bp and 20 bp), PA (237 bp, 217 bp, and 20 bp), and AA (237 bp). The size of the PCR amplification product for the Val290Met position of the PPAR-*γ* gene was 145 bp. Following digestion with the restriction endonuclease Nco I, the genotypes included GG (145 bp), GA (145 bp, 110 bp, and 35 bp), and AA (110 bp and 35 bp). The size of the PCR product for the Leu162Val position of the PPAR-*α* gene was 117 bp. Following digestion with the restriction endonuclease Hinf I, the genotypes included CC (117 bp), CG (117 bp, 94 bp, and 23 bp), and GG (94 bp and 23 bp). The size of the PCR product for the Gly482Ser position of the PGC-1*α* gene was 260 bp. Following digestion with the restriction endonuclease Hpa II, the genotypes included GG (149 bp and 111 bp), GA (260 bp, 149 bp, and 111 bp), and AA (260 bp). The PCR products for the Pro12Ala of PPAR-*γ* and Gly482Ser of PGC-1*α* gene were sent to BGI (Shanghai) for sequencing. The sequencing results are shown in Figures 1 and 2.

### 2.3. Physical Measurements

Height and weight were measured in all participants, and the mean of three measurements was used to calculate BMI (BMI = weight/height^2^ (kg/m^2^)). Blood pressure (BP) was measured using the brachial artery of the right arm (either in a seated position or in the mother's arms), and the process was repeated 2 min later. The mean value of both readings was recorded as the BP for each individual.

### 2.4. Blood Tests

Blood samples were taken from all participants following a bland meal the previous evening and an overnight fast. Fasting blood glucose (FBG), fasting serum insulin (FISN), fasting serum C-peptide (FCP), blood lipids, renal function, and coagulation were measured. All samples were obtained prior to steroid treatment. The kits for serum measurements and the 7600 automatic biochemical analyzer were provided by Hitachi (Japan). FISN and FCP were tested by chemiluminescence assays using kits and an Elecsys 2010 (Roche).

### 2.5. Calculation of HOMA-IR, Islet *β*-Cell Function, and Insulin Sensitivity Index

Homeostasis model assessment of insulin resistance (HOMA-IR), islet *β*-cell function (HOMA-islet), and insulin sensitivity index (ISI) were calculated using the following formulae: HOMA-IR = FBG × FISN/22.5; HOMA-islet = 20 × FISN/(FBG − 3.5); ISI = ln (1/FBG × FISN) [7] (FISN mU/L; FBG mmol/L).

### 2.6. Statistical Analyses

The group representation of the distribution of sample genotypes was estimated using the Hardy-Weinberg equilibrium. The frequency of each genotype was calculated using the gene-counting method. Normally distributed data were presented as mean ± standard deviation, and nonnormally distributed data were presented as median (range). The means of two groups with normal distributions were compared using *t*-tests or analysis of variance, and nonnormally distributed data were compared using non-parametric tests. Classified data were analyzed using *χ* ^2^ tests or logistic regression. Analyses were performed using SPSS 16.0 software, and *P*  values < 0.05 were considered statistically significant.

## 3. Results

### 3.1. Comparison of PPAR-*γ* (Pro12Ala) and PGC-1*α* (Gly482Ser) Genotypes between PNS and NC Groups

The frequencies of the PPAR-*γ* (Pro12Ala) and PGC-1*α* (Gly482Ser) genotypes in NC children conformed to the Hardy-Weinberg equilibrium. There were no significant differences in distributions of the PPAR-*γ* (Pro12Ala) and PGC-1*α* (Gly482Ser) genotypes between PNS and NC children (*P* > 0.99 and *P* = 0.324, resp.). The results are shown in Tables 1 and 2.

### 3.2. Comparison of Clinical Data between Individuals with Different Genotypes in the PNS Group

The results of a further-stratified analysis of the PPAR-*γ* (Pro12Ala) genotypes in PNS children are shown in Table 3. There were no significant differences in any of the following parameters among children with PNS with different PPAR-*γ* (Pro12Ala) genotypes: sex, age, BMI, levels of albumin (ALB), blood urea nitrogen (BUN), serum creatinine (Scr), blood uric acid (UA), GFR, total cholesterol (TC), triglycerides (TG), high-density lipoprotein (HDL-c), fibrinogen (Fbg), FBG, FCP, immunoglobulin G/M (IgG and IgM), complement C3 and C4, frequencies of CD3+ T cells, CD4+ T cells, CD8+ T cells, NK cells, and B lymphocytes, HOMA-islet values, and 24h urine protein (24-UP). However, patients with the PP genotype demonstrated significantly higher levels of FISN, IgA, and HOMA-IR and lower levels of ISI compared with patients with PA and AA genotypes.

The results of a further-stratified analysis of the PGC-1*α* (Gly482Ser) genotypes among PNS children are shown in Table 4. There were no significant differences in any of the following parameters among children with PNS with different PGC-1*α* (Gly482Ser) genotypes: sex, age, BMI, ALB, BUN, Scr, UA, GFR, TC, HDL-c, Fbg, IgA, IgG, IgM, FBG, FISN, FCP, and C4, frequencies of CD3+ T cells, CD4+ T cells, NK cells, and B lymphocytes, HOMA-IR, HOMA-islet, ISI values, and 24-UP. However, patients with the A allele demonstrated lower levels of CD8+ T cells and higher levels of TG and complement C3 than did patients with the G allele.

 The distribution of genes among children with PNS of different pathological types was analyzed by single-factor analysis of variance. There were no significant differences in PPAR-*γ* (Pro12Ala) and PGC-1*α* (Gly482Ser) genotypes between individuals with different renal pathological types. The results are shown in Tables 5 and 6.

 Gene distributions among children with PNS who demonstrated different responses to hormone treatment were analyzed by *χ* ^2^ tests and single-factor analysis of variance. There were no significant differences in PPAR-*γ* (Pro12Ala) and PGC-1*α* (Gly482Ser) genotypes among patients with different responses to hormone treatment. The results are shown in Tables 7 and 8.

## 4. Discussion

PPAR-*γ* receptors can be divided into three subtypes: *γ*l, *γ*2, and *γ*3. Several PPAR-*γ*2 mutations have been discovered, of which the most common is the CCA-GCA mutation at the 12th codon in exon 2, which results in the conversion of proline to alanine. This Pro12Ala gene polymorphism was first reported by Chung-Jen et al. [8] in a study of 34 Caucasian individuals. The mutation frequencies for Prol2Ala vary greatly among different ethnic groups: 12% in Caucasians, 10% in Americans, 4% in Japanese, and 2% in Chinese. Our study found the frequency of the A allele to be 3.8% and that of the P allele to be 96.2%. Other gene mutations of PPAR-*γ*2 (Prol15Gin, Val290Met, and Rro467Leu) exist at low frequencies and have smaller impacts on the population; however, the manifestations of these mutations include severe IR, local abnormal lipid metabolism, T2DM, and hypertension. The current study demonstrated that the Val290Met mutation in the PPAR-*γ*2 gene was absent in all 111 PNS and 111 NC children studied. Moreover, no patients in the current study suffered from severe IR, local abnormal lipid metabolism, T2DM, or hypertension; these observations support the rarity of this mutation. 

Previous studies showed an association between the Pro12Ala mutation and blood lipid levels, cardiovascular events, insulin sensitivity, T2DM, and renal function [2, 6, 9, 10]. Compared with NC children, children with PNS demonstrated increased BP, decreased GFR, increased Scr, hyperuricemia, hyperlipidemia, increased serum C-peptide levels, and decreased islet *β*-cell function during the early stage of the disease, though these patients did not demonstrate IR, a hypercoagulable state, or evidence of an immune inflammatory response. These results suggest that these characteristics may be correlated with the Pro12Ala gene polymorphism. We analyzed the distributions of two different genotypes in PNS and NC children and unexpectedly found no association between the Pro12Ala gene polymorphism and PNS occurrence. A further-stratified analysis in children with PNS showed that insulin levels and islet *β*-cell function were decreased and insulin sensitivity was increased in patients with the A allele, after matching for age, sex, and BMI. Stefan et al. [11] demonstrated that this mutation was associated with free fatty acid-induced reduction of second-phase insulin secretion in a healthy population with normal body weight. Furthermore, a large population study in Italian and Brazilian Caucasians showed that the A allele was significantly associated with increased insulin sensitivity [12], suggesting that the A allele may have distinct effects on insulin secretion and insulin sensitivity. However, the effects of the Pro12Ala polymorphism on insulin secretion and sensitivity remain inconclusive. Although most studies have shown that patients with mutations at this site have decreased insulin secretion capacity and increased insulin sensitivity, the specific mechanisms responsible for these effects remain unclear. 

 Previous studies confirmed that PPAR-*γ* participates in the pathophysiology of kidney diseases; its activation can reduce BP, delay renal arteriosclerosis, reduce proteinuria and Scr, and reverse the process of glomerulosclerosis and interstitial fibrosis, which are closely associated with prognosis in patients with kidney diseases. The Cys161Thr polymorphism of the PPAR-*γ*2 gene is associated with survival in patients with nonhypertensive IgA nephropathy [13]. We analyzed the association between the Pro12Ala polymorphism of the PPAR-*γ*2 gene and response to treatment in PNS patients and found no differences in genotype distributions between hormone-sensitive, hormone-resistant, and hormone-dependent patients. We were therefore unable to conclude that the Pro12Ala polymorphism of PPAR-*γ*2 could predict response to hormone treatment. Although no mutations in this gene were detected in children in the hormone-resistant group, this could have been because of the low mutation frequency of the A allele or the small sample size. In addition, the follow-up period in this study was short, and indicators such as progression to ESRD, survival rate, and complications could not be compared. Further follow-up studies are needed to address these factors. 

 Evidence has shown that PGC-1*α* is closely associated with MS and IR, and studies in different ethnic groups have found linkage between the chromosomal fragment of the PGC-1*α* gene and MS-related manifestations. Our study demonstrated that the frequency of the G allele was 46.6% and that of the A allele was 53.4%. Many previous studies have shown associations between the Gly482Ser polymorphism of the PGC-1*α* gene and IR, obesity, and type II diabetes [4]. Compared with NC children, children with PNS demonstrated increased BP, decreased GFR, increased Scr, hyperuricemia, hyperlipidemia, increased FCP, and decreased islet *β*-cell function at the time of disease onset, but did not have apparent IR, a hypercoagulable state, or evidence of an immune inflammatory response. We therefore speculated that this phenomenon might be associated with the PGC1*α* (Gly482Ser) gene polymorphism. We analyzed the distribution of three PGC1*α* genotypes in PNS and NC children and found no association between the PGC-1*α* (Gly482Ser) genotype and PNS occurrence. However, further-stratified analysis of children with PNS showed that blood TG levels in patients with the AA genotype were significantly increased after matching for age, sex, and BMI. Previous studies have shown that PGC-1*α* can collaborate with PPAR-*α*, PPAR-*γ*, and the farnesoid X receptor to enhance fatty acid oxidation and regulate TG metabolism [14]. Furthermore, mutations at the Gly482Ser position of the PGC-1*α* gene could also induce functional changes in PGC-1*α* [15]. These results suggest that functional changes in the PGC-1*α* gene resulting from the Gly482Ser mutation may affect TG metabolism. Animal studies demonstrated that liver TG levels in PGC-1*α*-knockout mice were increased threefold compared with the control group [16]. The results of the current study were consistent with those of previous studies, although differences in TG levels between genotypes were inconclusive. 

 We further analyzed glucose metabolism in PNS children and found no differences in FBG, FISN, FCP, HOMA-IR, HOMA-islet, and ISI among GG, GA, and AA genotypes. We were therefore unable to establish an association between the Gly482Ser site of the PGC-1*α* gene and glucose metabolism disorders and IR. However, several previous studies have demonstrated a close correlation between mutations at the Gly482Ser site of PGC-1*α* and glucose metabolism, and 482Ser was shown to increase the risk of type II diabetes in the population. Muller et al. [17] analyzed the SNPs of PGC-1*α* in nondiabetic Pima Indians and showed that the Gly482Ser site was associated with IR. Moreover, Pima Indians with the Gly/Gly genotype had lower levels of insulin secretion following glucose loading, suggesting that the PGC-1*α* gene could be a candidate gene for type II diabetes. This study found that patients with the Ser/Ser genotype had slightly higher FBG and HOMA-IR values and slightly lower FISN levels compared with patients with the other two genotypes, indicating that a correlation between the Gly482Ser site and glucose metabolism remains to be confirmed.

 In summary, the results of this study showed no significant correlations between the PPAR-*γ* (Prol2Ala) and PGC-1*α* (Gly482Ser) gene polymorphisms and the occurrence of PNS. However, mutation at the Pro12Ala position of the PPAR-*γ* gene may be associated with increased insulin sensitivity and decreased insulin secretion in children with PNS. Moreover, increased TG levels in patients with the PGC-1*α* (Gly482Ser) AA genotype suggest that this polymorphism may be responsible for TG abnormalities in children with PNS. 

## Figures and Tables

**Table 1:** The distribution of PPAR-*γ* (Pro12Ala) genotypes among PNS and NC children.

PPAR-*γ* genotype	PNS group		NC group		*χ* ^2^	OR (95% CI)	*P* value
	*n*	%	*n*	%			
PP	103	92.8	103	92.8	1.031	1	1.000
PA/AA	8	7.2	8	7.2		1.000 (0.312–2.766)	

**Table 2:** The distribution of PGC-1*α* (Gly482Ser) genotypes among PNS and NC children.

PGC-1*α* genotype	PNS group		NC group		*χ* ^2^	OR (95% CI)	*P* value
	*n*	%	*n*	%			
GG	18	16.7	20	18.2	2.253	1.037 (0.502–2.144)	0.324
GA	59	54.6	68	61.8		0.639 (0.276–1.478)	
AA	31	28.7	22	20.0		1	

**Table 3:** Stratified analysis of the PPAR-*γ* (Pro12Ala) genotypes among PNS children.

Subjects	PPAR-*γ* genotype		*t*/*t*′/*χ* ^2^/*z *	*P* value
	PP	PA/AA		
Gender (male/female)	74/29	6/2	0.000	1.000
Age (years)	3.67 (0.67–13.08)	3.00 (1.50–4.25)	−1.562	0.118
BMI	17.07 ± 0.22	17.24 ± 0.77	−0.205	0.838
sBP (kp)	14.28 ± 0.14	14.21 ± 0.29	0.120	0.905
dBP (kp)	9.16 ± 0.10	9.18 ± 0.37	−0.046	0.963
ALB (g/L)	17.70 (11.20–37.00)	18.95 (16.80–26.30)	−1.283	0.199
BUN (mmol/L)	4.00 (0.50–16.30)	2.60 (2.20–21.50)	−1.689	0.091
Scr (*μ*mol/L)	37.20 (18.30–207.60)	33.45 (27.60–51.50)	−1.032	0.302
UA (mmol/L)	350.76 ± 10.96	347.88 ± 34.60	0.071	0.943
GFR (mL/(min·1.73 m^2^))	137.54 ± 3.47	137.56 ± 8.75	−0.002	0.999
TC (mmol/L)	9.89 ± 0.28	9.37 ± 0.76	0.498	0.619
TG (mmol/L)	2.46 (0.77–8.03)	2.26 (1.36–6.33)	−0.160	0.873
HDL-c (mmol/L)	1.49 ± 0.06	1.46 ± 0.18	0.170	0.865
Fbg (g/L)	4.58 ± 0.16	3.37 ± 2.26	0.536	0.686
IgA (g/L)	1.06 ± 0.04	0.81 ± 0.08	2.701	0.020*
IgG (g/L)	2.26 (1.36–11.00)	2.31 (1.46–4.79)	−0.364	0.716
IgM (g/L)	1.70 ± 0.06	1.92 ± 0.27	−0.934	0.352
C3 (g/L)	2.49 ± 1.22	1.28 ± 0.06	0.279	0.781
C4 (g/L)	0.29 ± 0.01	0.27 ± 0.02	0.484	0.629
T (CD3+) (%)	68.95 ± 1.09	60.00 ± 7.11	1.700	0.093
T (CD4+) (%)	35.62 ± 0.96	31.25 ± 6.42	0.940	0.350
T (CD8+) (%)	27.92 ± 0.76	21.50 ± 3.95	1.764	0.081
NK (%)	8.00 (2.00–44.00)	5.00 (2.00–31.00)	−0.943	0.367
BC (%)	18.85 ± 0.87	23.75 ± 6.18	−1.156	0.251
FBG (mmol/L)	4.88 ± 0.06	4.84 ± 0.26	0.162	0.872
FISN (mU/L)	2.27 (0.20–16.63)	0.92 (0.20–3.17)	−2.526	0.012*
FCP (nmol/L)	0.47 (0.18–1.95)	0.41 (0.22–0.98)	−0.981	0.327
HOMA-IR	0.48 (0.04–4.31)	0.16 (0.05–0.82)	−2.566	0.060
HOMA-islet	38.06 (−141.54–895.56)	27.06 (2.50–90.94)	−1.460	0.144
ISI	−2.46 ± 0.10	−1.46 ± 0.33	−2.777	0.006**
24-h UP (mg/kg)	141.15 (2.96–1179.80)	117.84 (7.41–410.00)	−0.871	0.384

*P < 0.05, **P < 0.01.

**Table 4:** Stratified analysis of the PGC-1*α* (Gly482Ser) genotypes among PNS children.

Subjects	PGC-1*α* gene			*χ* ^2^/*F*	*P* value
	GA	AA	GG		
Gender (male/female)	46/13	21/10	11/7	2.388	0.303
Age (years)	3.60 (1.50–13.08)	3.00 (0.67–10.58)	4.40 (1.33–9.80)	3.058	0.217
BMI	16.84 ± 0.29	17.47 ± 0.36	16.98 ± 0.54	0.874	0.420
sBP (kp)	14.18 ± 0.17	14.36 ± 0.29	14.49 ± 0.34	0.384	0.682
dBP (kp)	9.15 ± 0.13	9.14 ± 0.18	9.19 ± 0.32	0.014	0.986
ALB (g/L)	17.60 (11.20–37.00)	18.10 (12.80–26.30)	18.40 (11.30–27.60)	0.327	0.849
BUN (mmol/L)	4.10 (2.30–21.50)	3.50 (0.50–16.30)	3.85 (2.20–13.10)	3.363	0.186
Scr (*μ*mol/L)	37.20 (18.30–67.80)	36.50 (23.00–207.60)	38.80 (25.30–69.70)	0.416	0.812
UA (mmol/L)	349.73 ± 14.53	357.90 ± 19.21	353.56 ± 27.52	0.055	0.946
GFR (mL/(min·1.73 m^2^))	139.62 ± 4.11	136.34 ± 7.52	135.69 ± 7.27	0.140	0.870
TC (mmol/L)	9.60 ± 0.36	10.12 ± 0.42	10.42 ± 0.89	1.164	0.559
TG (mmol/L)	2.60 ± 0.16	3.46 ± 0.30	2.77 ± 0.35	3.766	0.026*
HDL-c (mmol/L)	1.57 ± 0.08	1.38 ± 0.08	1.42 ± 0.11	1.299	0.277
Fbg (g/L)	4.32 ± 0.21	4.78 ± 0.42	4.79 ± 0.33	0.917	0.406
IgA (g/L)	1.03 ± 0.06	1.02 ± 0.08	1.10 ± 0.11	0.218	0.804
IgG (g/L)	2.25 (1.36–7.95)	2.30 (1.36–4.79)	1.96 (1.43–7.51)	0.044	0.978
IgM (g/L)	1.73 ± 0.09	1.75 ± 0.12	1.55 ± 0.12	0.603	0.549
C3 (g/L)	1.24 ± 0.03	1.36 ± 0.05	1.18 ± 0.07	3.193	0.045*
C4 (g/L)	0.28 (0.10–1.13)	0.28 (0.10–0.49)	0.26 (0.13–0.49)	0.412	0.814
T (CD3+) (%)	69.85 ± 1.25	64.62 ± 2.44	72.93 ± 2.13	4.755	0.093
T (CD4+) (%)	37.15 ± 1.26	33.77 ± 2.09	34.71 ± 1.50	1.312	0.275
T (CD8+) (%)	27.73 ± 0.88	25.19 ± 1.59	32.00 ± 1.86	4.469	0.014*
NK (%)	10.09 ± 1.04	10.77 ± 1.53	7.57 ± 0.94	1.012	0.368
BC (%)	17.68 ± 0.88	21.08 ± 2.00	18.07 ± 1.85	1.299	0.522
FBG (mmol/L)	4.82 ± 0.08	4.99 ± 0.13	4.85 ± 0.12	0.733	0.483
FISN (mU/L)	2.37 (0.20–9.43)	1.92 (0.57–16.63)	2.13 (0.76–12.87)	0.180	0.914
FCP (nmol/L)	0.45 (0.18–1.24)	0.50 (0.22–1.95)	0.50 (0.24–1.15)	2.386	0.303
HOMA-IR	0.68 ± 0.07	0.89 ± 0.19	0.76 ± 0.17	0.218	0.897
HOMA-islet	37.22 (−141.54–895.56)	38.47 (5.50–278.22)	28.78 (13.66–224.47)	0.192	0.908
ISI	−2.29 ± 0.14	−2.46 ± 0.18	−2.51 ± 0.19	0.468	0.628
24-h UP (mg/kg)	141.71 (11.25–1179.80)	125.88 (27.69–319.00)	176.52 (45.00–301.00)	1.064	0.587

*P < 0.05, **P < 0.01.

**Table 5:** The distribution of the PPAR-*γ* (Pro12Ala) genotypes among individuals with different renal pathological types.

PPAR-*γ* genotype	IgMN	MsPGN	IgAN	FSGS	C1qN	MCD	*χ* ^2^	*P* value
	*n* (%)	*n* (%)	*n* (%)	*n* (%)	*n* (%)	*n* (%)		
PP	5 (15.6%)	9 (28.1%)	3 (9.4%)	5 (15.6%)	5 (15.6%)	1 (3.1%)	5.698	0.285
PA/AA	2 (6.3%)	0 (0%)	2 (6.3%)	0 (0%)	0 (0%)	0 (0%)		

**Table 6:** The distribution of the PGC-1*α* (Gly482Ser) genotypes among individuals with different renal pathological types.

PGC-1*α* genotype	IgMN	MsPGN	IgAN	FSGS	C1qN	MCD	*χ* ^2^	*P* value
	*n* (%)	*n* (%)	*n* (%)	*n* (%)	*n* (%)	*n* (%)		
GG	2 (6.7%)	1 (3.3%)	1 (3.3%)	3 (10%)	0 (0%)	0 (0%)	11.633	0.252
GA	2 (6.7%)	6 (20%)	2 (6.7%)	1 (3.3%)	4 (13.3%)	0 (0%)		
AA	3 (10%)	1 (3.3%)	0 (0%)	2 (6.7%)	1 (3.3%)	1 (3.3%)		

**Table 7:** The distribution of the PPAR-*γ* (Pro12Ala) genotypes in groups with different responses to hormone treatment.

PPAR-*γ* genotype	Hormone-sensitive group	Hormone-resistant group	Hormone-dependent group	*χ* ^2^	*P* value
	*n* (%)	*n* (%)	*n* (%)		
PP	77 (71.3%)	13 (12.0%)	11 (10.2%)	0.779	0.824
PA/AA	6 (5.6%)	0 (0%)	1 (0.9%)		

**Table 8:** The distribution of the PGC-1*α* (Gly482Ser) genotypes in groups with different responses to hormone treatment.

PGC-1*α* genotype	Hormone-sensitive group	Hormone-resistant group	Hormone-dependent group	*χ* ^2^	*P* value
	*n* (%)	*n* (%)	*n* (%)		
GG	11 (10.5%)	3 (2.9%)	3 (2.9%)	3.500	0.473
GA	47 (44.8%)	6 (5.7%)	4 (3.8%)		
AA	24 (22.2%)	5 (4.8%)	2 (1.9%)		
